# Folate, Vitamin B_6_, and Vitamin B_12_ Status in Association With Metabolic Syndrome Incidence

**DOI:** 10.1001/jamanetworkopen.2022.50621

**Published:** 2023-01-11

**Authors:** Jie Zhu, Cheng Chen, Liping Lu, James M. Shikany, Mary E. D’Alton, Ka Kahe

**Affiliations:** 1 Nutrition and Foods Program, School of Family and Consumer Sciences, Texas State University, San Marcos; 2 Department of Obstetrics and Gynecology, Vagelos College of Physician and Surgeons, Columbia University Irving Medical Center, New York, New York; 3 Department of Epidemiology, Mailman School of Public Health, Columbia University Irving Medical Center, New York, New York; 4 Division of Preventive Medicine, School of Medicine, University of Alabama at Birmingham, Birmingham

## Abstract

**Importance:**

The associations of B vitamin status with metabolic syndrome (MetS) incidence among the US population remain unclear.

**Objective:**

To investigate intakes and serum concentrations of folate, vitamin B_6_, and vitamin B_12_ in association with MetS risk in a large US cohort.

**Design, Setting, and Participants:**

This prospective study included Black and White young adults in the US who were enrolled from 1985 to 1986 and studied until 2015 to 2016. Diet was assessed using a validated diet history at examination years 0, 7, and 20. Serum concentrations of folate, vitamin B_6_, and vitamin B_12_ were assayed at examination years 0, 7, and 15 in a subset of 1430 participants. MetS was ascertained by clinic and laboratory measurements and self-reported medication use. Data were analyzed between January and July 2021.

**Exposures:**

Intakes and serum levels of folate, vitamin B_6_, and vitamin B_12_.

**Main Outcomes and Measures:**

Multivariable Cox proportional hazards regression models were used to calculate hazard ratios (HRs) and 95% CIs for the associations of energy-adjusted B vitamin intakes or serum B vitamin levels with incident MetS.

**Results:**

The study included 4414 participants, with 2225 Black individuals (50.4%) and 2331 women (52.8%). The mean (SD) age at baseline was 24.9 (3.6) years. A total of 1240 incident MetS cases occurred during the 30 years (mean [SD], 22.1 [9.5] years) of follow-up. Compared with the lowest quintile of each energy-adjusted B vitamin intake, the HRs for incident MetS in the highest quintile were 0.39 (95% CI, 0.31-0.49) for folate (*P *for trend < .001), 0.61 (95% CI, 0.46-0.81) for vitamin B_6_ (*P *for trend = .002), and 0.74 (95% CI, 0.58-0.95) for vitamin B_12_ (*P *for trend = .008) after adjustment for potential confounders. Similarly, significant inverse associations were observed in the subset with serum data on these B vitamins (folate: HR, 0.23; 95% CI, 0.17-0.33; *P *for trend < .001; vitamin B_6_: HR, 0.48; 95% CI, 0.34-0.67; *P *for trend < .001; and vitamin B_12_: HR, 0.70; 95% CI, 0.51-0.96; *P *for trend = .01).

**Conclusions and Relevance:**

This prospective cohort study found that intakes and serum concentrations of folate, vitamin B_6_, and vitamin B_12_ were inversely associated with incident MetS among Black and White young adults in the US.

## Key Points

**Question:**

Are intakes and serum levels of folate, vitamin B_6_, and vitamin B_12_ associated with metabolic syndrome (MetS) incidence among the US population?

**Findings:**

In this cohort study of 4414 US adults, intakes and serum concentrations of folate, vitamin B_6_, and vitamin B_12_ were inversely associated with incident MetS among Black and White young adults in the US.

**Meaning:**

These findings suggest that adequate intakes of folate, vitamin B_6_, and vitamin B_12_ should be recommended for prevention of MetS.

## Teaser

This cohort study evaluates the association between folate, vitamin B_6_, and vitamin B_12_ status and metabolic syndrome incidence among US adults.

## Introduction

Folate, vitamin B_6_, and vitamin B_12_ are essential nutrients for nucleic acid synthesis and methyl group generation.^1^ Folate from diet or folic acid (FA) from supplements can donate a carbon group to homocysteine (Hcy), which can be either methylated into methionine or degraded into cysteine, with vitamins B_6_ and B_12_ serving as essential coenzymes.^2^ Emerging evidence suggests that low status of these B vitamins may lead to adiposity, dyslipidemia, vascular endothelial dysfunction, glucose intolerance, and insulin resistance,^3,4,5,6,7^ which have been implicated in the pathogenesis of metabolic syndrome (MetS).

Data directly linking these B vitamins to incident MetS are sparse. A few randomized clinical trials (RCTs) found that B vitamin supplementation alleviated MetS-associated components. FA supplementation alone or with vitamin B_6_ and/or vitamin B_12_ reduced blood pressure (BP),^8,9^ improved insulin resistance,^10^ and had favorable outcomes on lipid metabolic profiles.^11,12^ However, these studies did not examine MetS risk directly and concentrated on an elderly cohort or populations at high risk for cardiovascular diseases. Thus, the generalizability of the results to the general population remains to be determined. To the best of our knowledge, data remain unavailable on the longitudinal association of these B vitamin intakes with the development of MetS among the general population of adults in the US.

To provide further evidence, the hypotheses that higher intakes and serum concentrations of folate, vitamin B_6_, and vitamin B_12_ would be prospectively associated with lower incident MetS among Black and White young adults in the US were examined in the present analysis, using data from the Coronary Artery Risk Development in Young Adults (CARDIA) study.

## Methods

### Study Population

The CARDIA study enrolled Black and White adults in the US aged 18 to 30 years from 4 US metropolitan cities (Birmingham, Alabama; Chicago, Illinois; Minneapolis, Minnesota; and Oakland, California) from 1985 to 1986 (year 0).^13^ The CARDIA study includes Black and White participants to let researchers explore racial inequities in cardiovascular disease risk factors and outcomes. Race was assessed by self-report at baseline and confirmed at exam year 2 in the CARDIA study.^14^ The CARDIA study included Black and White participants because it let researchers explore racial inequities in cardiovascular disease risk factors and outcomes. To date, 8 follow-up examinations have been conducted at years 2, 5, 7, 10, 15, 20, 25, and 30, respectively. This cohort study was approved by the institutional review board at each study center. All participants provided written informed consent.

Participants were excluded if they had implausible total energy intake (50 participants with ≤600 or ≥6000 kcal per day for women or ≤800 or ≥8000 kcal per day for men), had no vitamin B intake data (2 participants), fasted less than 8 hours before blood drawing at any examination (16 participants), were pregnant at any examination (230 participants), had MetS or whose MetS status could not be determined at baseline (254 participants), or had insufficient information to ascertain MetS in follow-up examinations (148 participants) (eFigure in Supplement 1). This study followed the Strengthening the Reporting of Observational Studies in Epidemiology—Nutritional Epidemiology (STROBE-nut) reporting guideline.

### Ascertainment of MetS

Detailed protocols and manuals of operations for clinic measurements have been previously reported.^15^ Briefly, waist circumference was examined at the minimum abdominal girth in duplicate when the participant was standing.^15^ BP was measured on each participant’s right arm 3 times at 1-minute intervals after a 5-minute rest. The mean of the second and third examinations was used in the analysis.^4^ Blood samples were drawn after at least 8-hours of fasting. Plasma concentrations of triglyceride and high-density lipoprotein cholesterol (HDL-C) were measured using enzymatic methods.^16^ Additionally, fasting plasma glucose concentrations were assayed by the hexokinase ultraviolet method at baseline and a radioimmunoassay from examination year 7 to year 30, with these 2 examinations recalibrated to ensure comparability.^17^

MetS components were defined according to the diagnosis and management of the MetS established by the American Heart Association and National Heart, Lung, and Blood Institute.^18^ Participants with at least 3 of the individual MetS components were defined to have MetS. Incident cases of MetS were identified at each follow-up examination.

### Dietary Assessment

Dietary information was assessed using the validated, interviewer-administered CARDIA diet history at the baseline, year 7, and year 20 examinations.^13,19^ Nutrient consumption was computed via the Nutrition Data System for Research (version 10, 20, and 36 at the baseline, year 7, and year 20, respectively). This study assessed each B vitamin intake from both dietary and supplemental sources. Total folate consumption was measured at baseline and follow-ups at year 7 and 20 visits, whereas intakes of vitamin B_6_ and vitamin B_12_ were assessed at year 7 and 20. The overall dietary quality was estimated by calculating the a priori diet quality scores at baseline, year 7, and year 20.^20^ To best represent long-term intake habits and to decrease within-participant variation, we calculated the means of B vitamin intakes, total energy intake, and the a priori diet quality scores from baseline before the time of incident MetS or MetS components, or last follow-up, whichever came first, and used them in the analyses.

### Assessment of Serum B Vitamins and Homocysteine Concentration

Fasting serum samples were collected in a subcohort of 1430 participants at examination years 0, 7, and 15 and stored at −70 °C until analysis. Serum folate and vitamin B_12_ were determined on the Hitachi 911 (Roche Diagnostics) with the use of the CEDIA homogeneous enzyme immunoassay system (Boehringer Mannheim). Serum vitamin B_6_ was examined using a radioenzymatic assay (American Laboratory Products).^4^ Serum Hcy was examined by a fluorescence polarization immunoassay (IMx Homocysteine Assay; Axis Biochemicals ASA) via the IMx analyzer (Abbott Diagnostics).^4^ The accumulative mean serum levels before the incident MetS during the follow-up were used in the analyses.

### Covariates

A set of verified self-administered questionnaires were adopted to collect participants’ information including sociodemographics and medical and lifestyle factors at baseline and each follow-up.^13^ Participants were classified as never, former, or current smokers at the time of incident outcome or the end of follow-up. Alcohol intake was measured and converted to milliliters per day.^21^ Physical activity during the previous 12 months was measured by the CARDIA physical activity questionnaire, with physical activity score calculated in exercise units.^22^ Cumulative mean alcohol consumption and physical activity from baseline to incident outcome or the end of follow-up were used in the analyses. Participants’ weight and height at baseline were measured in duplicate, and means were calculated, which were used to calculate body mass index (BMI, calculated as weight in kilograms divided by height in meters squared) at baseline. Family histories of diabetes, hypertension, or myocardial infarction were identified as either mother or father having these diseases.

### Statistical Analysis

Participants were divided into quintiles according to B vitamin intakes or serum concentrations. Characteristics of participants were described as means or percentages. The median value was provided for variables with skewed distribution. The differences of baseline characteristics across energy-adjusted B vitamin intake quintiles were compared by using analysis of variance, χ^2^ test, or Kruskal-Wallis test, as appropriate. Cox proportional hazards regression was used to examine the association between each energy-adjusted B vitamin intake and MetS incidence. The energy-adjusted B vitamin intakes were calculated as the B vitamin intakes per day divided by the total daily calorie intake. Multivariable-adjusted hazard ratios (HRs) and corresponding 95% CIs were calculated for the second to the fifth quintiles of energy-adjusted B vitamin intakes, using the lowest quintile as the reference. The medians of energy-adjusted B vitamin quintiles were used as a continuous variable to test the linear trend. We performed the analysis in 2 sequential multivariable-adjusted models. In Model 1, we adjusted for age, sex, race, CARDIA field center, and total energy intake. In Model 2 (final model), we further adjusted for education, lifestyle factors (smoking status, alcohol consumption, PA, use of B vitamin supplement of interest, a priori diet quality scores), and family medical history (hypertension, diabetes, and heart attack).

We stratified the analysis by some prespecified factors that are closely associated with MetS, including age, sex, and race as well as supplement usage. The interaction terms between the medians of B vitamin intake quintiles and these potential outcome modifiers were created and tested for statistical significance. Furthermore, we examined the associations between each B vitamin intake and individual component of MetS using Cox proportional hazards regression with covariates in the final model. The *P* values were adjusted for multiple comparisons using the Bonferroni procedure.

We performed several sensitivity analyses. First, we additionally adjusted for the other 2 B vitamin intakes for each B vitamin intake to reduce the potential confounding. Second, we identified the participants with low intakes of all 3 energy-adjusted B vitamins (those with intakes less than the median levels) and compared the incidence of MetS among this subgroup with the rest.

We calculated the Spearman correlation between the average intake of total folate, vitamin B6, or vitamin B12 and their serum mean concentration in this subcohort. We also examined the associations of serum B vitamin quintiles with MetS incidence using Cox proportional hazards regression and adjusted for covariates in the final model except for the a priori diet quality scores.

Finally, to explore potential mechanisms, we examined the Spearman correlations between serum concentrations of folate, vitamin B_6_, and B_12_ with Hcy. We also examined the association between Hcy quintiles and MetS incidence in a Cox proportional hazards regression model with adjustment for covariates in the final model except for the a priori diet quality scores.

All analyses were performed by using SAS version 9.4 (SAS Institute Inc). 2-Sided tests were used, and *P* ≤ .05 was considered significant for main outcomes and interactions. Data analysis was conducted from January to July 2021.

## Results

The present prospective cohort study includes 4414 participants with 2225 (50.4%) Black individuals and 2331 (52.8%) women. The mean (SD) age at baseline was 24.9 (3.6) years (Table 1). A total of 1240 incident MetS cases occurred during the 30 years (mean [SD], 22.1 [9.5] years) of follow-up. Participants who had higher intakes of total folate, vitamin B_6_, or vitamin B_12_ were more likely to be older, be women and White, have a higher education level, be never-smokers, have less alcohol consumption (for folate and vitamin B_6_), be more active, have lower total energy intake, and better overall dietary quality. They were also less likely to have family history of diabetes (for folate). In addition, they had lower BMI, lower systolic BP, lower waist circumference, lower triglycerides (for folate and vitamin B_12_), higher HDL-C, and lower serum Hcy at baseline (Table 1).

**Table 1.** Baseline Characteristics of the Study Population by Quintiles of Energy-Adjusted B Vitamin Intakes, the CARDIA study, 1985 to 2015^a^^,^^b^

Characteristic	Total participants, No.	Folate (μg/1000 kcal)			B_6_ (mg/1000 kcal)			B_12_ (μg/1000 kcal)		
		Participants, No. (%)		*P* value	Participants, No. (%)		*P* value	Participants, No. (%)		*P* value
		Quintile 1	Quintile 5		Quintile 1	Quintile 5		Quintile 1	Quintile 5	
Patients, No.	4414	882	882		754	754		754	754	
Level, median (IQR)	NA	91.9 (79.4-101.6)	353.7 (303.1-456.1)	NA	0.7 (0.6-0.7)	2.8 (2.1-4.4)	NA	1.4 (1.2-1.6)	8.2 (6.4-12.6)	NA
Supplemental use^c^	NA	256 (29.0)	798 (90.5)	<.001	48 (6.4)	737 (97.8)	<.001	60 (8.0)	684 (90.7)	<.001
Corresponding serum levels, median (IQR), μg/dL	NA	9.7 (5.3-13.6)	17.9 (13.1-24.0)	<.001	6.4 (3.4-11.4)	16.3 (8.7-46.8)	<.001	0.4 (0.3-0.6)	0.5 (0.4-0.7)	<.001
Age, mean (SD), y	24.9 (3.6)	23.9 (3.8)	25.6 (3.4)	<.001	24.2 (3.8)	25.4 (3.5)	<.001	24.7 (3.8)	25.5 (3.4)	<.001
Sex										
Female	2331 (52.8)	401 (45.5)	631 (71.5)	<.001	359 (47.6)	493 (65.4)	<.001	404 (53.6)	455 (60.3)	<.001
Male	2083 (47.2)	481 (54.5)	251 (28.5)	<.001	395 (52.4)	261 (34.6)	<.001	350 (46.4)	299 (39.7)	<.001
Race										
Black	2225 (50.4)	621 (70.4)	294 (33.3)	<.001	496 (65.8)	293 (38.9)	<.001	432 (57.3)	310 (41.1)	<.001
White	2189 (49.6)	261 (29.6)	588 (66.7)	<.001	258 (34.2)	461 (61.1)	<.001	322 (42.7)	444 (58.9)	<.001
Education levels, median (IQR), y	14.0 (12.0-16.0)	13.0 (12.0-15.0)	16.0 (14.0-18.0)	<.001	14.0 (12.0-16.0)	16.0 (14.0-18.0)	<.001	14.0 (12.0-16.0)	16.0 (14.0-18.0)	<.001
Smoking status										
Never	2570 (58.8)	412 (46.9)	591 (67.8)	<.001	377 (50.3)	490 (65.5)	<.001	423 (56.5)	473 (63.4)	<.001
Former	876 (20.1)	138 (15.7)	199 (22.8)		134 (17.9)	181 (24.2)		146 (19.5)	184 (24.7)	
Current	923 (21.1)	328 (37.4)	82 (9.4)		238 (31.8)	77 (10.3)		180 (24.0)	89 (11.9)	
Alcohol consumption, median (IQR), ml/d	5.7 (0.9-15.9)	6.5 (0.9-20.3)	4.3 (0.8-11.9)	<.001	5.8 (0.8-16.2)	4.6 (0.8-12.4)	<.001	4.9 (0.6-15.8)	5.6 (0.9-14.0)	.15
Physical activity, median (IQR), exercise units	327.6 (201.3-493.7)	287.7 (159.7-453.5)	350.9 (227.8-512.6)	<.001	270.3 (156.0-424.2)	360.7 (227.6-527.7)	<.001	296.0 (180.4-455.9)	360.7 (225.0-529.6)	<.001
Total energy intake, mean (SD), kcal/d	2772.4 (1171.4)	3229.4 (1425.0)	2228.5 (817.5)	<.001	2992.0 (1218.9)	2354.0 (899.4)	<.001	2830.2 (1131.2)	2434.5 (932.2)	<.001
A priori dietary quality scores	61.8 (11.1)	52.1 (7.7)	70.1 (9.6)	<.001	54.2 (8.3)	68.5 (10.0)	<.001	60.0 (11.0)	67.4 (9.9)	<.001
Family history of diabetes	611 (13.8)	139 (15.8)	91 (10.3)	.01	120 (15.9)	89 (11.8)	.14	94 (12.5)	90 (11.9)	.14
Family history of hypertension	2197 (49.8)	453 (51.4)	426 (48.3)	.16	382 (50.7)	369 (48.9)	.90	397 (52.7)	368 (48.8)	.40
Family history of heart attack	623 (14.1)	126 (14.3)	122 (13.8)	.94	103 (13.7)	104 (13.8)	.78	102 (13.5)	100 (13.3)	.29
Body mass index at Y0, mean (SD)^d^	24.3 (4.7)	25.1 (5.4)	23.9 (4.4)	<.001	24.7 (5.4)	23.9 (4.2)	<.001	24.5 (5.1)	23.8 (4.2)	.048
Glucose at Y0, mean (SD), mg/dL	82.2 (13.0)	82.0 (8.6)	81.5 (10.5)	.18	82.1 (9.7)	81.4 (11.1)	.11	82.0 (9.5)	82.0 (14.2)	.39
Blood pressure at Y0, mean (SD), mm Hg										
Systolic	110.3 (10.7)	111.5 (10.6)	108.5 (10.6)	<.001	111.0 (10.9)	109.0 (10.4)	<.001	110.6 (11.1)	109.2 (10.3)	.02
Diastolic	68.5 (9.2)	68.6 (9.7)	68.2 (8.7)	.06	68.4 (9.6)	68.2 (8.7)	.20	68.8 (9.7)	68.6 (8.9)	.16
Waist circumstance at Y0, mean (SD), cm	77.3 (10.7)	79.6 (12.1)	75.1 (9.9)	<.001	78.6 (11.8)	75.5 (9.9)	<.001	77.8 (11.1)	75.8 (9.9)	<.001
Triglycerides at Y0, mean (SD), mg/dL	70.4 (43.4)	73.4 (41.6)	66.9 (34.2)	.008	70.1 (37.8)	67.0 (37.5)	.30	69.0 (36.3)	65.8 (32.8)	.03
HDL-C at Y0, mean (SD), mg/dL	53.3 (13.0)	50.7 (12.7)	55.6 (12.8)	<.001	51.7 (12.5)	55.3 (13.0)	<.001	52.9 (13.0)	55.4 (12.9)	<.001
Serum homocysteine, mean (SD), mg/dL	1.2 (0.5)	1.3 (0.4)	1.1 (0.3)	<.001	1.3 (0.3)	1.1 (0.3)	<.001	1.4 (0.8)	1.1 (0.5)	<.001

Abbreviations: CARDIA, The Coronary Artery Risk Development in Young Adults; HDL-C, high-density lipoprotein cholesterol; NA, not applicable; Y, CARDIA exam year.

^a^ Results are presented by mean (SD), median (IQR), or proportions.

^b^ *P* values are for any difference across quintiles of energy-adjusted B vitamin intake levels by using analysis of variance, Kruskal-Wallis test, or χ^2^ test as appropriate.

^c^ Supplemental use means the use of each B vitamin supplement.

^d^ Body mass index is calculated as weight in kilograms divided by height in meters squared.

### Intakes of Folate, Vitamin B_6_, and Vitamin B_12_ and MetS

After adjustment for potential confounders, participants in the highest quintile of energy-adjusted total folate intake had 61% lower incidence of MetS compared with those in the lowest intake group (HR, 0.39; 95% CI, 0.31-0.49; *P *for trend < .001 in Model 2) (Table 2). Similarly, the incidence of MetS was 39% lower for vitamin B_6_ (HR, 0.61; 95% CI, 0.46-0.81; *P *for trend = .002) and 26% lower for vitamin B _12_ intake (HR, 0.74; 95% CI, 0.58-0.95; *P *for trend = .008] in Model 2 (Table 2).

**Table 2.** Multivariable-Adjusted HRs (95% CIs) of Incident Metabolic Syndrome by Quintiles of Energy-Adjusted B Vitamin Intake Levels, the CARDIA Study, 1985 to 2015^a^^,^^b^^,^^c^

Characteristic	**Energy-adjusted nutrient intake**					*P* value for linear trend
	Quintile 1	Quintile 2	Quintile 3	Quintile 4	Quintile 5	
Folate						
Range, μg/1000 kcal	<111.4	111.4-148.1	148.1-194.1	194.1-272.6	≥272.6	NA
Median, μg/1000 kcal	91.9	129.3	168.7	226.8	353.7	NA
Cases No./total No.	307/882	289/883	251/883	209/883	184/882	NA
Model 1, HR (95% CI)	1 [Reference]	0.70 (0.59-0.82)	0.54 (0.45-0.64)	0.40 (0.34-0.49)	0.35 (0.28-0.42)	<.001
Model 2, HR (95% CI)	1 [Reference]	0.72 (0.61-0.85)	0.57 (0.47-0.69)	0.43 (0.35-0.54)	0.39 (0.31-0.49)	<.001
Vitamin B_6_						
Range, μg/1000 kcal	<0.8	0.8-0.9	0.9-1.2	1.2-1.8	≥1.8	NA
Median, μg/1000 kcal	0.7	0.8	1.0	1.4	2.8	NA
Cases No./total No.	267/754	253/755	219/754	188/755	178/754	NA
Model 1, HR (95% CI)	1 [Reference]	0.94 (0.79-1.11)	0.77 (0.64-0.93)	0.62 (0.51-0.76)	0.58 (0.47-0.70)	<.001
Model 2, HR (95% CI)	1 [Reference]	1.02 (0.86-1.22)	0.88 (0.71-1.08)	0.65 (0.50-0.84)	0.61 (0.46-0.81)	.002
Vitamin B_12_						
Range, μg/1000 kcal	<1.8	1.8-2.4	2.4-3.3	3.3-5.4	≥5.4	NA
Median, μg/1000 kcal	1.4	2.1	2.8	4.1	8.2	NA
Cases No./total No.	232/754	262/755	220/754	210/755	181/754	NA
Model 1, HR (95% CI)	1 [Reference]	1.11 (0.93-1.32)	0.88 (0.73-1.05)	0.83 (0.69-1.01)	0.67 (0.55-0.82)	<.001
Model 2, HR (95% CI)	1 [Reference]	1.08 (0.90-1.30)	0.88 (0.72-1.07)	0.84 (0.67-1.04)	0.74 (0.58-0.95)	.008

Abbreviations: CARDIA, The Coronary Artery Risk Development in Young Adults; HR, hazard ratio.

^a^ Cox proportional hazard regression models were used. Linear trend was examined by using the medians of energy-adjusted B vitamin quintiles as a continuous variable.

^b^ Model 1 was adjusted for age, sex (female or male), race (White or Black), study center, and total energy intake (continuous).

^c^ Model 2 was additionally adjusted for education levels (<12, 12-15.9, ≥16 years), smoking status (never, former, or current smokers), alcohol consumption (0, 0.1-11.9, 12-23.9, ≥24 ml/d), physical activity levels (quintiles), supplement use of B vitamin of interest (yes or no), family histories of diabetes, hypertension, and heart attack (all yes or no), and a priori diet quality score (continuous).

### Serum Folate, Vitamin B_6_, and Vitamin B_12_ and MetS

B vitamin intakes were significantly correlated with their serum concentrations among 1430 participants with available data (*R* = 0.309 for folate, 0.312 for vitamin B_6_, and 0.212 for vitamin B_12_, all *P* < .001). Serum concentrations of the 3 B vitamins were inversely associated with incident MetS (quintile 5 vs 1: HR, 0.23; 95% CI, 0.17-0.33; *P *for trend < .001 for folate; HR, 0.48; 95% CI, 0.34-0.67; *P *for trend < .001 for vitamin B_6_; HR, 0.70; 95% CI, 0.51-0.96; *P *for trend = .01 for vitamin B_12_) (Table 3). Serum concentrations of folate, vitamin B_6_, and vitamin B_12_ were also inversely correlated with serum Hcy concentration. The corresponding Spearman partial correlation coefficients were −0.43, −0.22, and −0.37, respectively (all *P* < .001). Moreover, serum higher Hcy was associated with an increased incidence of MetS (HR in quintile 2-5 vs 1: 1.45; 95% CI, 1.06-1.98; HR, 1.15; 95% CI, 0.82-1.62; HR, 1.42; 95% CI, 1.02-2.00; HR, 2.00; 95% CI, 1.44-2.79; *P *for trend < .001) (eTable 2 in Supplement 1).

**Table 3.** Multivariable-Adjusted HRs (95% CIs) of Incident Metabolic Syndrome by Quintiles of Serum B Vitamin Concentrations Among 1430 Participants, the CARDIA Study, 1985 to 2015^a^

Characteristic	Serum B vitamins					*P* value for linear trend
	Quintile 1	Quintile 2	Quintile 3	Quintile 4	Quintile 5	
Serum folate						
Median level (IQR), μg/dL	5.60 (3.80-7.00)	10.05 (9.10-11.00)	14.30 (13.10-15.10)	17.50 (16.70-18.80)	26.00 (23.00-29.70)	<.001
Cases No./total No.	155/287	100/286	93/285	50/281	47/288	
Fully adjusted, HR (95% CI)^a^	1 [Reference]	0.51 (0.39-0.66)	0.47 (0.36-0.61)	0.26 (0.18-0.36)	0.23 (0.17-0.33)	
Serum vitamin B_6_						
Median level (IQR), μg/dL	2.57 (1.56-3.36)	5.89 (4.99-6.77)	9.96 (8.77-11.27)	16.73 (14.50-20.39)	47.33(34.47-71.29)	<.001
Cases No./total No.	121/285	119/286	83/287	63/285	59/286	
Fully adjusted, HR (95% CI)^a^	1 [Reference]	0.96 (0.73-1.24)	0.69 (0.51-0.93)	0.54 (0.39-0.75)	0.48 (0.34-0.67)	
Serum vitamin B_12_						
Median level (IQR), μg/dL	0.29 (0.25-0.33)	0.40 (0.38-0.41)	0.48 (0.46-0.50)	0.59 (0.56-0.64)	0.84 (0.75, 0.96)	.01
Cases No./total No.	95/286	102/284	85/286	88/285	75/285	
Fully adjusted, HR (95% CI)^a^	1 [Reference]	1.04 (0.79-1.39)	0.82 (0.61-1.11)	0.86 (0.64-1.16)	0.70 (0.51-0.96)	

Abbreviations: CARDIA, The Coronary Artery Risk Development in Young Adults; HR, hazard ratio.

^a^ Cox proportional hazard regression models were used. Linear trend was examined by using the medians of B vitamin quintiles as a continuous variable. Models were adjusted for covariates in Model 2 (Table 2) except the a priori diet quality scores.

### Stratified and Sensitivity Analyses

In the stratified analyses by age groups (<vs ≥ median 25 years at baseline), sex, race (Black vs White) and supplementation (users and nonusers), the inverse associations between B vitamin intakes and MetS incidence persisted across subgroups except that the association between vitamin B_12_ and MetS existed only among supplement users (eTable 1 in Supplement 1). In addition, total folate intake was inversely associated with the incidences of all individual components of MetS (Table 4). Significant inverse associations were found between vitamin B_6_ intake and all individual components except for high glucose, and between vitamin B_12_ intake and all individual components except for high glucose as well as high BP (Table 4). These significant associations remained when adjusted for multiple comparisons.

**Table 4.** Multivariable-Adjusted HRs (95% CIs) of Individual Components of Metabolic Syndrome by Quintiles of Energy-Adjusted B Vitamin Intake Levels, the CARDIA Study, 1985 to 2015^a^

Characteristic	Cases No./total No.	Hazard ratio (95% CI)					*P* value for linear trend
		Quintile 1	Quintile 2	Quintile 3	Quintile 4	Quintile 5	
Folate							
High glucose	906/4334	1 [Reference]	0.81 (0.66-0.99)	0.72 (0.58-0.89)	0.59 (0.46-0.76)	0.47 (0.36-0.63)	<.001
High BP	1833/4090	1 [Reference]	0.68 (0.59-0.78)	0.55 (0.47-0.65)	0.45 (0.38-0.54)	0.49 (0.41-0.60)	<.001
Abdominal obesity	1857/4126	1 [Reference]	0.81 (0.70-0.93)	0.61 (0.52-0.71)	0.50 (0.42-0.60)	0.46 (0.38-0.55)	<.001
High triglycerides	1268/4198	1 [Reference]	0.73 (0.62-0.86)	0.52 (0.43-0.62)	0.44 (0.36-0.55)	0.48 (0.38-0.60)	<.001
Low HDL-C	1356/3331	1 [Reference]	0.66 (0.56-0.77)	0.46 (0.38-0.55)	0.32 (0.26-0.39)	0.25 (0.20-0.31)	<.001
Vitamin B_6_							
High glucose	823/3730	1 [Reference]	1.11 (0.91-1.37)	1.00 (0.79-1.27)	0.96 (0.71-1. 29)	0.78 (0.55-1.08)	.04
High BP	1605/3474	1 [Reference]	0.98 (0.84-1.14)	0.85 (0.72-1.02)	0.83 (0.67-1.02)	0.75 (0.60-0.94)	.03
Abdominal obesity	1647/3500	1 [Reference]	1.03 (0.89-1.20)	0.86 (0.73-1.02)	0.75 (0.62-0.92)	0.64 (0.52-0.80)	<.001
High triglycerides	1128/3610	1 [Reference]	1.19 (0.99-1.43)	0.91 (0.74-1.12)	0.72 (0.56-0.92)	0.68 (0.52-0.89)	.005
Low HDL-C	1201/2853	1 [Reference]	1.07 (0.90-1.28)	0.95 (0.78-1.14)	0.70 (0.56-0.87)	0.57 (0.45-0.74)	<.001
Vitamin B_12_							
High glucose	823/3730	1 [Reference]	0.99 (0.80-1.22)	1.16 (0.93-1.45)	1.25 (0.98-1.60)	1.02 (0.77-1.36)	.81
High BP	1605/3474	1 [Reference]	1.14 (0.97-1.32)	1.02 (0.87-1.20)	0.97 (0.81-1.16)	0.88 (0.73-1.07)	.04
Abdominal obesity	1647/3500	1 [Reference]	0.88 (0.76-1.02)	0.85 (0.73-1.00)	0.77 (0.65-0.91)	0.70 (0.58-0.85)	<.001
High triglycerides	1128/3610	1 [Reference]	1.03 (0.87-1.24)	0.77 (0.63-0.93)	0.67 (0.54-0.84)	0.68 (0.54-0.86)	.002 < .01
Low HDL-C	1201/2853	1 [Reference]	0.82 (0.69-0.97)	0.69 (0.58-0.82)	0.60 (0.49-0.73)	0.48 (0.38-0.60)	<.001

Abbreviations: BP, blood pressure; CARDIA, The Coronary Artery Risk Development in Young Adults; HDL-C, high-density lipoprotein cholesterol; HR, hazard ratio.

^a^ Cox proportional hazard regression models were used. Linear trend was examined by using the medians of energy-adjusted B vitamin quintiles as a continuous variable. Models were adjusted for covariates in Model 2 (Table 2) plus baseline levels of each corresponding individual component.

The results of the sensitivity analyses were generally consistent. First, when we further adjusted for the other 2 B vitamin intakes, the association between each B vitamin intake and MetS incidence did not appreciably change (eTable 3 in Supplement 1). Second, comparing those with low intakes of all 3 energy-density B vitamins with the rest, the risk of developing MetS was significantly higher (HR, 2.67; 95% CI, 2.02-3.53; *P *for trend < .001).

## Discussion

In this large longitudinal cohort study with 30-years of follow-up, total intakes of folate, vitamin B_6_, and vitamin B_12_ were significantly inversely associated with incidence of MetS and the majority of its individual components among adults in the US. Serum concentrations of these B vitamins were inversely associated with incident MetS in a subset of these participants.

Epidemiological data on dietary B vitamin and MetS risk have produced inconsistent results. A cross-sectional analysis using data from the National Health and Nutrition Examination Survey showed that higher dietary intakes of folate and vitamin B_6_, but not vitamin B_12_, were related to lower prevalence of MetS among US adult civilians.^23^ However, another cross-sectional survey found null associations between these B vitamin intakes and incident MetS among urban Iranian adults.^24^ A prospective cohort study of American Indian tribal members aged 14 years or older found that intake of vitamin B_6_, but not folate, was inversely associated with incident MetS over a median 5.3 years of follow-up.^2^ The discrepancy of results in these studies may be partly explained by different MetS ascertainment methods, diverse ethnic backgrounds, different frequency of dietary intake measurements, and varying follow-up periods.

In addition, a meta-analysis including 12 RCTs reported that high-dose (≥5000 μg per day) FA supplementation for 2 to 16 weeks significantly reduced systolic BP.^8^ Another RCT found that Hcy-lowering treatment with FA (5 mg per day) plus vitamin B_6_ (250 mg per day) reduced BP in a clinically healthy population.^9^ Likewise, other RCTs demonstrated that FA supplementation alone or with vitamin B_12_ and vitamin B_6_ improved insulin resistance^12^ and had beneficial outcomes on lipid metabolic profiles.^11,12^

We found that folate intake was inversely associated with all individual components of MetS. However, the inverse association between vitamin B_6_ and MetS may be mainly explained by its favorable outcomes on adiposity, hypertriglyceridemia, low HDL-C, and elevated BP. Regarding vitamin B_12_, its inverse association with MetS may be mainly explained by its favorable impacts on adiposity, hypertriglyceridemia, and low HDL-C. Moreover, results of stratified analyses manifested that age (<vs ≥ median 25 years at baseline), sex, and race (Black vs White) did not materially modify the inverse associations between these B vitamin intakes and incidental MetS. Nevertheless, the inverse association between vitamin B_12_ intake and MetS risk was only observed among supplement users, presumably because these participants consumed relatively higher levels of total vitamin B_12_.

The robustness of our findings is supported by the consistent results of intakes and serum concentrations of these B vitamins. The findings of serum B vitamin concentrations are consistent with a case-control study that reported lower plasma vitamin B_6_ concentrations in Nigerian MetS patients^25^ and cross-sectional studies that found inverse associations between blood FA,^26^ vitamin B_6,_^27^ and vitamin B_12,_^26,28^ and MetS prevalence in various populations.

Our findings are biologically plausible. Emerging evidence suggests that folate, vitamin B_6_, and B_12_ are essential for facilitating energy, lipid, and carbohydrate metabolism in rodents and humans,^3,29,30,31^ mainly by counteracting hyperhomocysteinemia.^3,6^ Our exploratory analysis showed inverse association between serum B vitamin and serum Hcy concentrations, and positive association of serum Hcy with MetS incidence. However, when we adjusted for serum Hcy in Model 2, the observed B vitamins-MetS associations were slightly attenuated but remained significant, suggesting that it is likely to be a multifactorial mechanism. For example, low status of these B vitamins may lead to dyslipidemia,^3^ vascular endothelial dysfunction,^4,5^ glucose intolerance,^6^ and insulin resistance^7^ through oxidative stress, systemic inflammation, and impairment of nitric oxide synthesis,^3,6,32,33,34^ which have been implicated in the pathogenesis of MetS. In addition, folate donates the methyl group in the one-carbon cycle with vitamin B_6_ and B_12_ acting as cofactors, which modifies methylation status of genetic loci signals related to cellular insulin resistance, elevated BP, and adiposity.^30,31^ However, whether the association between these B vitamins and incident MetS in humans can be attributed to the specific DNA methylation modification involved in lipid and glucose metabolism warrants future investigation.

### Strengths and Limitations

This study had notable strengths. First, the study population includes a large community-based cohort of young adults fairly balanced across sex and race. The long-term longitudinal follow-up, comprehensive data collection for exposure, outcome and covariates, and the stringent quality control in the CARDIA study allow us to examine the prospective associations of interest independent of major MetS factors associated with risk and to explore potential outcome modification. Second, MetS components were ascertained by measures obtained by trained personnel using standardized procedures, rather than self-report, which substantially reduces the possibility of measurement error and misdiagnosis of end points. Third, we performed comprehensive analyses examining B vitamins measured in both diet and serum, and their associations with MetS as well as its components. The consistent results suggest that it is unlikely that our results were substantially biased.

There are limitations that merit discussions. First, although we adjusted for a wide array of potential confounders, residual confounding cannot be completely ruled out due to the nature of observational study. Hence, this study cannot establish a causal inference. Folate-rich foods, such as green leafy vegetables, fruit, and legumes contain other beneficial nutrients and compounds that may confound the observed inverse associations. However, we adjusted for the a priori diet quality scores as a marker of overall dietary quality. The consistent findings for serum and dietary B vitamins also suggest the observed association is less likely to be explained by confounding. Second, we did not examine the potential outcomes of genetic risk factors influencing B vitamin metabolism. Previous studies reported relations between genetic variations that encode the key enzymes in B vitamin metabolism and incident MetS.^35,36,37^ The gene-diet interaction may affect the bioavailable amount of B vitamins and modify the B vitamins-MetS association, thus warranting further investigation.

## Conclusions

In this study, we found that both dietary intakes and serum concentrations of folate, vitamin B_6_, and vitamin B_12_ were significantly inversely associated with incident MetS. Adequate intakes of these B vitamins should be recommended for prevention of MetS. Lower serum concentrations of these B vitamins may be indicators of higher risk of MetS. Further investigations are needed to confirm our findings and establish causal inference.

## Notes

Supplement 1. **eFigure. **Flowchart of the Coronary Artery Risk Development in Young Adults (CARDIA) Study Participants Included in This Study **eTable 1. **Stratified Analyses for Dietary B Vitamins in Relation to Incident Metabolic Syndrome, the CARDIA Study, 1985 to 2015 **eTable 2. **Association of Serum Homocysteine Levels With Incident Metabolic Syndrome, the CARDIA Study, 1985 to 2015 **eTable 3. **Multivariable-Adjusted HRs (95% CIs) of Incident Metabolic Syndrome by Quintiles of Energy-Adjusted B Vitamin Intake Levels, the CARDIA Study, 1985 to 2015—Additional Adjustment for the Other 2 B Vitamins

Supplement 2.
Data Sharing Statement
