# Supplementary material for: Folate, Vitamin B6, and Vitamin B12 Status in Association With Metabolic Syndrome Incidence
Source: JAMA Netw Open. 2023 Jan 11;6(1):e2250621. doi: 10.1001/jamanetworkopen.2022.50621 (PMC9856626; doi:10.1001/jamanetworkopen.2022.50621)
Supplement: Supplement 1. — eFigure. Flowchart of the Coronary Artery Risk Development in Young Adults (CARDIA) Study Participants Included in This Study eTable 1. Stratified Analyses for Dietary B Vitamins in Relation to Incident Metabolic Syndrome, the CARDIA Study, 1985 to 2015 eTable 2. Association of Serum Homocysteine Levels With Incident Metabolic Syndrome, the CARDIA Study, 1985 to 2015 eTable 3. Multivariable-Adjusted HRs (95% CIs) of Incident Metabolic Syndrome by Quintiles of Energy-Adjusted B Vitamin Intake Levels, the CARDIA Study, 1985 to 2015—Additional Adjustment for the Other 2 B Vitamins [file jamanetwopen-e2250621-s001.pdf]

Supplemental Online Content

Zhu J, Chen C, Lu L, Shikany JM, D'Alton ME, Kahe K. Folate, vitamin B_6, and vitamin B_{12} status in association with metabolic syndrome incidence. *JAMA Netw Open*. 2023;6(1):e2250621. doi:10.1001/jamanetworkopen.2022.50621

eFigure. Flowchart of the Coronary Artery Risk Development in Young Adults (CARDIA) Study Participants Included in This Study

eTable 1. Stratified Analyses for Dietary B Vitamins in Relation to Incident Metabolic Syndrome, the CARDIA Study, 1985 to 2015

eTable 2. Association of Serum Homocysteine Levels With Incident Metabolic Syndrome, the CARDIA Study, 1985 to 2015

eTable 3. Multivariable-Adjusted HRs (95% CIs) of Incident Metabolic Syndrome by Quintiles of Energy-Adjusted B Vitamin Intake Levels, the CARDIA Study, 1985 to 2015—Additional Adjustment for the Other 2 B Vitamins

This supplemental material has been provided by the authors to give readers additional information about their work.

© 2023 Zhu J et al. *JAMA Network Open.*

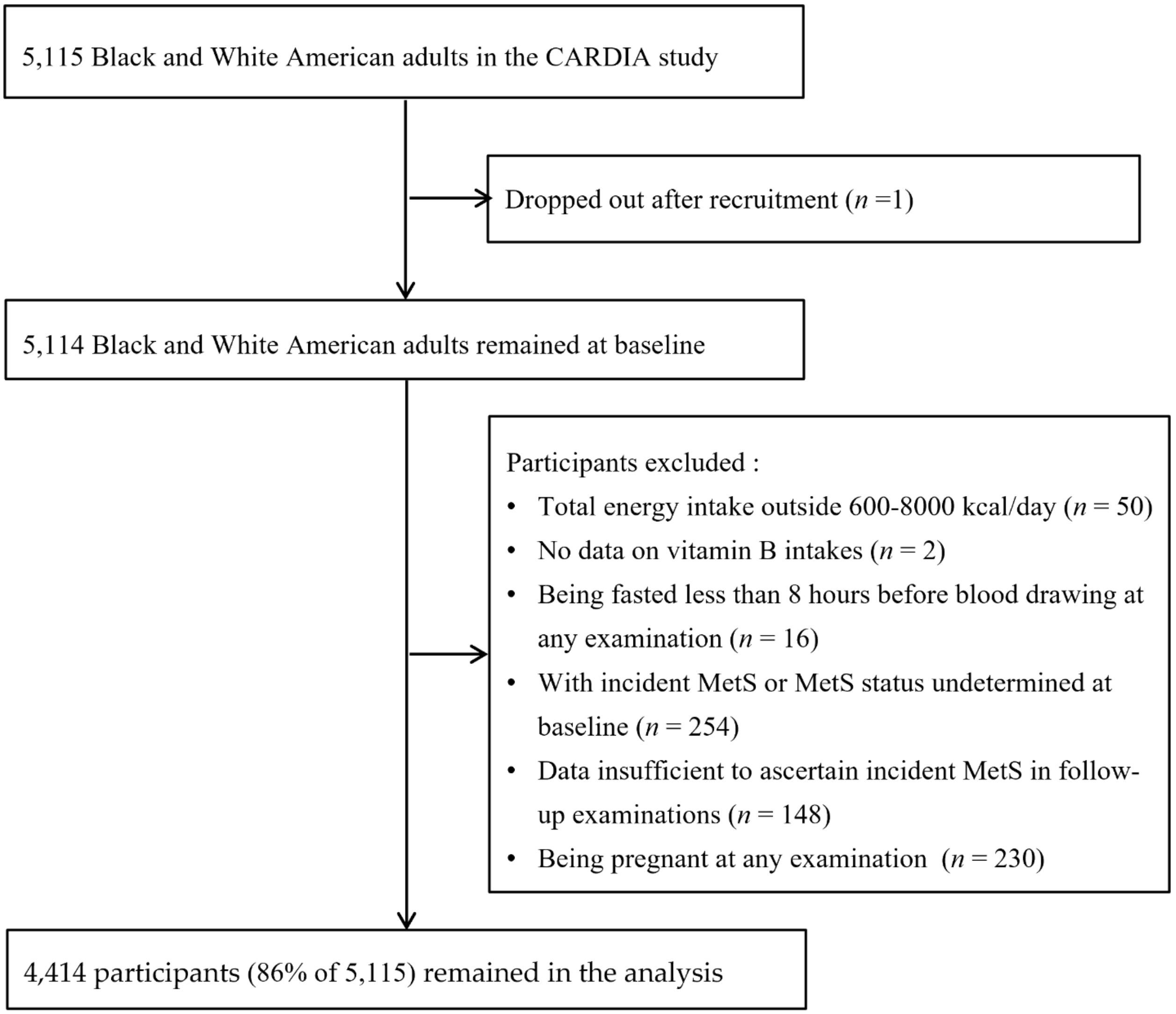

eFigure. Flowchart of the Coronary Artery Risk Development in Young Adults (CARDIA) Study Participants Included in This Study

© 2023 Zhu J et al. *JAMA Network Open.*

eTable 1. Stratified Analyses for Dietary B Vitamins in Relation to Incident Metabolic Syndrome, the CARDIA Study, 1985 to 2015[a]

	Hazard ratio (95% CI)					
	Quintiles of nutrient intake					
Characteristic	Quintile 1	Quintile 2	Quintile 3	Quintile 4	Quintile 5	*P* value for linear trend
Folate (n = 4413)						
Range (µg/1000 kcal)	<111.4	111.4-148.1	148.1-194.1	194.1-272.6	≥272.6	-
Median (µg/1000 kcal)	91.9	129.3	168.7	226.8	353.7	-
Cases No. /total No.	307/882	289/883	251/883	209/883	184/882	-
Age ≤ 25 y	1 (Reference)	0.69 (0.54-0.88)	0.62 (0.48-0.81)	0.53 (0.39-0.71)	0.43 (0.30-0.61)	<.001
Age > 25 y	1 (Reference)	0.71 (0.56-0.91)	0.50 (0.38-0.65)	0.35 (0.26-0.48)	0.34 (0.25-0.48)	<.001
P for interaction = 0.04						
Female	1 (Reference)	0.86 (0.67-1.09)	0.70 (0.54-0.92)	0.53 (0.39-0.71)	0.49 (0.36-0.67)	<.001
Male	1 (Reference)	0.61 (0.49-0.78)	0.46 (0.35-0.60)	0.36 (0.26-0.49)	0.35 (0.24-0.51)	<.001
P for interaction = 0.90						
Black	1 (Reference)	0.76 (0.61-0.94)	0.63 (0.49-0.80)	0.53 (0.40-0.70)	0.46 (0.33-0.64)	<.001
White	1 (Reference)	0.59 (0.45-0.77)	0.44 (0.33-0.59)	0.32 (0.23-0.45)	0.33 (0.23-0.47)	<.001
P for interaction = 0.03						
Supplement users	1 (Reference)	0.66 (0.51-0.85)	0.45 (0.35-0.58)	0.38 (0.29-0.50)	0.35 (0.26-0.47)	<.001
Supplement non-users	1 (Reference)	0.74 (0.59-0.93)	0.72 (0.55-0.94)	0.47 (0.31-0.71)	0.47 (0.25-0.88)	<.001
P for interaction = 0.87						
Vitamin B_6 (n = 3772)						
Range (µg/1000 kcal)	<0.8	0.8-0.9	0.9-1.2	1.2-1.8	≥1.8	-
Median (µg/1000 kcal)	0.7	0.8	1.0	1.4	2.8	-
Cases No. /total No.	267/754	253/755	219/754	188/755	178/754	-
Age ≤ 25 y	1 (Reference)	1.07 (0.84-1.38)	0.88 (0.65-1.19)	0.78 (0.54-1.12)	0.74 (0.50-1.12)	.21
Age > 25 y	1 (Reference)	0.95 (0.73-1.23)	0.84 (0.63-1.13)	0.53 (0.36-0.77)	0.50 (0.34-0.74)	.004
P for interaction = 0.11						
Female	1 (Reference)	1.06 (0.81-1.38)	1.21 (0.91-1.62)	0.86 (0.60-1.24)	0.88 (0.60-1.30)	.28
Male	1 (Reference)	0.94 (0.73-1.20)	0.61 (0.45-0.83)	0.48 (0.33-0.70)	0.41 (0.27-0.63)	.001
P for interaction = 0.85						
Black	1 (Reference)	1.13 (0.89-1.42)	0.95 (0.72-1.26)	0.75 (0.52-1.08)	0.74 (0.50-1.10)	.16

© 2023 Zhu J et al. *JAMA Network Open.*

(continued)

eTable 1. Stratified analyses for dietary B vitamins in relation to incident metabolic syndrome, the CARDIA study, 1985-2015[a] (continued)

	Hazard ratio (95% CI)					
	Quintiles of nutrient intake					
Characteristic	Quintile 1	Quintile 2	Quintile 3	Quintile 4	Quintile 5	*P* value for linear trend
White	1 (Reference)	0.87 (0.66-1.16)	0.79 (0.58-1.09)	0.55 (0.37-0.80)	0.51 (0.34-0.78)	.012
P for interaction = 0.33						
Supplement users	1 (Reference)	0.42 (0.26-0.69)	0.23 (0.15-0.35)	0.18 (0.12-0.27)	0.20 (0.14-0.30)	.007
Supplement non-users	1 (Reference)	1.06 (0.87-1.30)	1.07 (0.84-1.36)	1.22 (0.85-1.76)	0.36 (0.09-1.47)	.66
P for interaction = 0.11						
Vitamin B_{12} (n = 3772)						
Range (µg/1000 kcal)	<1.8	1.8-2.4	2.4-3.3	3.3-5.4	≥5.4	-
Median (µg/1000 kcal)	1.4	2.1	2.8	4.1	8.2	-
Cases No. /total No.	232/754	262/755	220/754	210/755	181/754	-
Age ≤ 25 y	1 (Reference)	1.21 (0.94-1.58)	1.02 (0.77-1.35)	1.12 (0.81-1.53)	0.92 (0.65-1.30)	.31
Age > 25 y	1 (Reference)	0.97 (0.75-1.25)	0.74 (0.56-0.97)	0.63 (0.46-0.86)	0.60 (0.43-0.84)	.009
P for interaction = 0.19						
Female	1 (Reference)	1.14 (0.88-1.48)	0.86 (0.65-1.14)	0.90 (0.67-1.22)	0.91 (0.65-1.27)	.59
Male	1 (Reference)	1.04 (0.80-1.34)	0.85 (0.64-1.11)	0.77 (0.56-1.07)	0.57 (0.40-0.83)	.001
P for interaction = 0.38						
Black	1 (Reference)	1.23 (0.97-1.56)	0.99 (0.76-1.28)	0.92 (0.68-1.24)	0.85 (0.61-1.19)	.16
White	1 (Reference)	0.84 (0.64-1.11)	0.68 (0.51-0.92)	0.70 (0.50-0.97)	0.59 (0.42-0.84)	.02
P for interaction = 0.33						
Supplement users	1 (Reference)	0.53 (0.33-0.83)	0.34 (0.22-0.51)	0.33 (0.22-0.49)	0.31 (0.21-0.46)	.002
Supplement non-users	1 (Reference)	1.14 (0.93-1.39)	1.00 (0.80-1.25)	1.15 (0.86-1.53)	1.13 (0.71-1.77)	.57
P for interaction = 0.01						

Abbreviations: CARDIA, The Coronary Artery Risk Development in Young Adults; IQR, inter-quartile range.

[a] Cox proportional hazard regression models were used. Linear trend was examined by using the medians of B vitamin quintiles as a continuous variable. Models were adjusted for age, sex (female or male), race (white or black), study center, total energy intake (continuous), education levels (<12, 12-15.9, ≥16 years), smoking status (never, former, or current smokers), alcohol consumption (0, 0.1-11.9, 12-23.9, ≥24 ml/day), physical activity levels (quintiles), supplement use of B vitamin of interest (yes or no), and family histories of diabetes, hypertension, and heart attack (all yes or no).

© 2023 Zhu J et al. *JAMA Network Open.*

eTable 2. Association of Serum Homocysteine Levels With Incident Metabolic Syndrome, the CARDIA Study, 1985 to 2015[a]

	Hazard ratio (95% CI)					
	Quintiles of homocysteine					
Model	Quintile 1	Quintile 2	Quintile 3	Quintile 4	Quintile 5	*P* value for linear trend
Homocysteine (n = 1430)						
Range (mg/dL)	<0.95	0.95-1.10	1.10-1.23	1.23-1.45	≥1.45	
Median (mg/dL)	0.85	1.01	1.15	1.31	1.65	
Cases No. /total No.	70/292	95/293	74/269	89/288	118/288	
Fully Adjusted[a]	1 (Reference)	1.45 (1.06-1.98)	1.15 (0.82-1.62)	1.42 (1.02-2.00)	2.00 (1.44-2.79)	<.001

Abbreviations: CARDIA, The Coronary Artery Risk Development in Young Adults.

[a] Cox proportional hazard regression model was used. Linear trend was examined by using the medians of homocysteine quintiles as a continuous variable. [a]Models were adjusted for age, sex (female or male), race (white or black), study center, total energy intake (continuous), education levels (<12, 12-15.9, ≥16 years), smoking status (never, former, or current smokers), alcohol consumption (0, 0.1-11.9, 12-23.9, ≥24 ml/day), physical activity levels (quintiles), supplement use of B vitamin of interest (yes or no), and family histories of diabetes, hypertension, and heart attack (all yes or no).

© 2023 Zhu J et al. *JAMA Network Open.*

eTable 3. Multivariable-Adjusted HRs (95% CIs) of Incident Metabolic Syndrome by Quintiles of Energy-Adjusted B Vitamin Intake Levels, the CARDIA Study, 1985 to 2015—Additional Adjustment for the Other 2 B Vitamins[a,b]

	Hazard ratio (95% CI)					
	Quintiles of energy-adjusted nutrient intake					
Characteristic	Quintile 1	Quintile 2	Quintile 3	Quintile 4	Quintile 5	*P* value for linear trend
Folate (n = 4413)						
Range (µg/1000 kcal)	<111.4	111.4-148.1	148.1-194.1	194.1-272.6	≥272.6	-
Median (µg/1000 kcal)	91.9	129.3	168.7	226.8	353.7	-
Cases No. /total No.	307/882	289/883	251/883	209/883	184/882	-
Model 2 + Vitamin B_6 and B_{12}	1 (Reference)	0.65 (0.54-0.78)	0.51 (0.42-0.63)	0.41 (0.33-0.52)	0.37 (0.29-0.47)	<.001
Vitamin B_6 (n = 3772)						
Range (µg/1000 kcal)	<0.8	0.8-0.9	0.9-1.2	1.2-1.8	≥1.8	-
Median (µg/1000 kcal)	0.7	0.8	1.0	1.4	2.8	-
Cases No. /total No.	267/754	253/755	219/754	188/755	178/754	-
Model 2 + folate and Vitamin B_{12}	1 (Reference)	1.02 (0.85-1.22)	0.88 (0.71-1.08)	0.64 (0.49-0.83)	0.59 (0.44-0.78)	<.001
Vitamin B_{12} (n = 3772)						
Range (µg/1000 kcal)	<1.8	1.8-2.4	2.4-3.3	3.3-5.4	≥5.4	-
Median (µg/1000 kcal)	1.4	2.1	2.8	4.1	8.2	-
Cases No. /total No.	232/754	262/755	220/754	210/755	181/754	-
Model 2 + folate and Vitamin B_6	1 (Reference)	1.08 (0.90-1.30)	0.88 (0.72-1.06)	0.83 (0.67-1.04)	0.73 (0.57-0.94)	.006

Abbreviations: CARDIA, The Coronary Artery Risk Development in Young Adults; CI, confidence interval; HR, hazard ratio;.

[a] Cox proportional hazard regression models were used. Linear trend was examined by using the medians of energy-adjusted B vitamin quintiles as a continuous variable.

[b] Models were adjusted for covariates in Model 2 (Table 2) plus the other two energy-adjusted B vitamin intakes (continuous) for each B vitamin.

© 2023 Zhu J et al. *JAMA Network Open.*
