# Supplementary material for: Folate, Vitamin B6, and Vitamin B12 Status in Association With Metabolic Syndrome Incidence
Source: JAMA Netw Open. 2023 Jan 11;6(1):e2250621. doi: 10.1001/jamanetworkopen.2022.50621 (PMC9856626; doi:10.1001/jamanetworkopen.2022.50621)
Supplement: Supplement 2. — Data Sharing Statement [file jamanetwopen-e2250621-s002.pdf]

Data Sharing Statement

Zhu. Folate, Vitamin B_6, and Vitamin B_{12} Status in Association With Metabolic Syndrome Incidence. *JAMA Netw Open*. Published January 11, 2023.
doi:10.1001/jamanetworkopen.2022.50621

Data
Data available: No

Additional Information
Explanation for why data not available: The present study is a secondary data analysis study, so data access and sharing will not be available until the CARDIA study committee approves it.
